# Psychometric properties of an Arabic translation of the meaning in life scale in a sample of young adults

**DOI:** 10.1186/s12888-024-05776-2

**Published:** 2024-04-24

**Authors:** Roni Chaaya, Ecem Yakın, Diana Malaeb, Rabih Hallit, Sahar Obeid, Feten Fekih-Romdhane, Souheil Hallit

**Affiliations:** 1 Social and Education Sciences Department, School of Arts and Sciences, Lebanese American University, Jbeil, Lebanon; 2 Centre d’Études et de Recherches en Psychopathologie et Psychologie de la Santé, Université de Toulouse-Jean Jaurès, UT2J, 5 allées Antonio Machado, Toulouse, 31058 France; 3 College of Pharmacy, Gulf Medical University, Ajman, United Arab Emirates; 4 School of Medicine and Medical Sciences, Holy Spirit University of Kaslik, P.O. Box 446, Jounieh, Lebanon; 5 Department of Infectious Disease, Notre Dame, Secours University Hospital Center, Street 93, Postal Code 3 Byblos, Lebanon; 6 Department of Infectious Disease, Bellevue Medical Center, Mansourieh, Lebanon; 7 The Tunisian Center of Early Intervention in Psychosis, Department of Psychiatry “Ibn Omrane”, Razi hospital, Manouba, 2010 Tunisia; 8 Faculty of Medicine of Tunis, Tunis El Manar University, Tunis, Tunisia; 9 Psychology Department, College of Humanities, Effat University, Jeddah, 21478 Saudi Arabia; 10 Applied Science Research Center, Applied Science Private University, Amman, Jordan

**Keywords:** Meaning in life, MLQ, Young adults, Arabic, Psychometric properties

## Abstract

**Background:**

Young adults are in a constant phase of realizing their meaning in life while being in a constant pursuit of meaning. Meaning in life is a subjective, personal construct related to the perception of one’s own life. Considering that there are no measures that study this construct within the Arab context, this study aimed to examine the psychometric properties of an Arabic translation of the Meaning in Life Questionnaire (MLQ) in the Lebanese context with a sample of young adults.

**Methods:**

A sample of 684 Lebanese young adults was recruited for this study, having a mean age of 21.74 years, 65.6% of which were females. Through an online questionnaire, participants were requested to complete the Meaning in Life Questionnaire (MLQ), Depression, Anxiety and Stress Scale (DASS-8) and the Oviedo Grit Scale (EGO).

**Results:**

CFA indicated that fit of the original bi-dimensional model of MLQ scores was inadequate. Items 9 and 10 cross-loaded to both MLQ factors. After removal of those 2 items, the final model displayed good fit indices. Reliability was good for the Search (ω = 0.89 / α = 0.89) and Presence (ω = 0.88 / α = 0.87) subscales. Additionally, across three levels of gender invariance (Configural, Metric and Scalar), no significant gender-based distinctions were observed in the MLQ scores. The Search subscale was significantly and positively associated with higher GRIT but not psychological distress, whereas the Presence subscale was significantly associated with higher GRIT and lower psychological distress.

**Conclusion:**

The results of this study contribute to the psychometric reliability and validity of the Arabic version of the MLQ and makes it available for dissemination among young adults within the Arab context. This allows for the implementation of new research that target construct of meaning in life, allowing for the accessibility of interventions that aim to foster the presence of and search for meaning in the lives of young adults within the Arab nations.

## Introduction

There are various ways to understand the concept of meaning in life. Steger et al., for instance, referred to it as one’s personal perception and importance of life, as well as the experience of being and existing from an individual perspective [1]. Others, however, described the concept of meaning in life as a deep sense of significance that arises from evaluating one’s life as purposeful and meaningful with a sense of direction and belonging [2]. In order to achieve meaning in their lives, individuals might seek to fulfil their desires or needs for purpose, self-worth, efficacy, and value [3]. Conversely, others expressed that meaning in life can be understood as a complex cognitive system that impacts one’s coherence and purpose in life, encompassing the pursuit as well as the attainment of personal goals [4]. While there are varying definitions of meaning in life, there is consensus that its role is vital as a motivating factor in enhancing individual well-being [5].

In 1963, Victor Frankl proposed that humans possess an inherent “will to meaning” that is characterized as an intrinsic motivation to seek meaning and purpose in one’s life [1]. For Frankle, the search for meaning in life is the driving motivational factor in human beings [6]. The failure to achieve this meaning in life correlated with higher levels of psychological distress [1], with a higher need of therapy being associated with a reduced sense of meaning in life [7]. This is concordant with the fact that a lack of meaning in life is linked to higher rates of depression, anxiety, suicidal ideation and substance abuse [1, 8]. Conversely, Individuals who experience higher levels of meaning in life tend to express greater enjoyment in their work, increased life satisfaction, and overall happiness [1]. In accordance with the preceding information, having greater meaning in life was associated with less distress and a lower likelihood of having repetitive negative thinking [9]. Studies have also demonstrated ‘meaning in life’ as being a predictor of global psychological wellbeing, purpose in life and self-acceptance [10]. It has also been shown that seeking meaning in life serves as a protector against emotional volatility and enables psychological health and wellbeing [11].

Although a number of scales measure the concept of meaning in life, such as the Purpose in Life Test (PIL [12]), the Life regard Index (LRI [13]), and the Sense of Coherence Scale [14], these scales tend to cause confusion within the framework of meaning [1]. More specifically, the scales have faced criticism due to having items that confound with variables other than that of meaning in life, such as items that tackle mood instead [1]. Moreover, the ‘search for meaning’ variable, although given much attention since its advent in Frankl’s Man’s Search for Meaning in 1963, it has been mostly neglected in the instruments that measure meaning in life [1].

In order to offset these drawbacks, a group of researchers aimed to develop the Meaning in Life Questionnaire (MLQ) that defines meaning in life “as the sense made of, and significance felt regarding, the nature of one’s being and existence”, based on Frankl’s ideology that people uniquely develop their life’s meaning [1]. Three studies were conducted for the development of the MLQ; the first study focused on the 44-item MLQ which resulted from a confirmatory factor analysis (CFA) of 83 sample items. Through a Scree-plot analysis, six factors emerged, with two dominant factors, namely: the presence of meaning / purpose in one’s life, and the search for meaning. Both factors constituted 17 out of the 44 items. Through a goodness of fit analysis, the two-factor model did not achieve acceptable fit with the 17 items. This led to the removal of 3 items which were leading to the reduction of the model fit. The model that showed the best fit is that of a 10-item MLQ with 5 items related to each of the two factors, showing good internal consistency and convergent validity [1].

The second study was conducted to further prove the association between the two factors of the MLQ found in the first study, proved strong factor loadings, and showed good fit of the model [1]. The third study conducted by the researchers aimed to further study the subscale’s convergent and discriminant validities, as well as compare their discriminant validities to other scales that measure meaning [1]. The MLQ Presence (MLQ-P) (α = 0.82) and MLQ Search (MLQ-S) (α = 0.87) were seen to have good reliability, with the third study giving support to the convergent and discriminant validities of both subscales [1].

The MLQ has been validated across a variety of populations, with the Hindi translation of the two-factor scale (MLQ-H) showing psychometrically sound properties with MLQ Presence having a Cronbach’s α = 0.81, and the MLQ Search showing a Cronbach’s α = 0.78 [15]. The Italian version of the MLQ showed similar results, proving through a factor analysis the appropriateness of the two-factor structure of the MLQ within the Italian population, adding proof to its internal consistency (MLQ-P: α = 0.84; MLQ-S: α = 0.90), convergent and discriminant validities [16]. A Turkish version of the scale (MLQ-TR) also exists, showing an internal consistency of α = 0.88 for the MLQ-P and α = 0.90 for the MLQ-S, proving support for the scale’s convergent validity in the Turkish context; however, a negative association between the two factors was seen among the tested sample [17]. However, to our knowledge, there appears to be no Arabic version of the MLQ.

### The present study

This study aims to explore the psychometric properties of the MLQ scale within a sample of Arabic-speaking Lebanese young adults. Studies have proven that having meaning in life can aid in the mitigation of negative consequences of psychological health problems that individuals may experience during adulthood [18]. Having a clear understanding of one’s meaning in life contributes to psychological wellbeing, especially in young adults who are in a transitional stage trying to figure out their new roles and shape their own paths as they become adults [19]. The presence of a sense of meaning in life has been correlated with improvements in physical and mental health, occupational and social lifestyles, and a lengthier lifespan. Therefore, comprehending this construct in young adults would enable its fostering during this age [19]. However, there remains a gap in the literature regarding the applicability of these findings to culturally diverse populations. Considering that cultural and ethnic differences exist when it comes to studying the construct of meaning in life [20], it would be efficient to study the reliability and validity of the MLQ and the meaning in life construct in different cultures and population [1], like Lebanon, for example. By focusing on Lebanese young adults, we aim to contribute to the broader comprehension of meaning in life within a unique cultural context. Lebanon has been impacted by the rise in globalization and the surge in cross-cultural interactions, resulting in a multicultural environment within the nation [21]. This renders it an intriguing context for exploring how the validation of the MLQ fares in such a culturally diverse setting. This assertion holds particular significance noting that the cultural milieu in which individuals grow shapes their perspectives on deriving meaning from life, profoundly impacting their perceptions and comprehension of the world around them [22]. Additionally, considering that other translated versions of the MLQ have given support to the two-factor structure of the MLQ [15–17], it is expected to find concordant results with the Arabic version of the MLQ within a Lebanese sample of young adults.

## Methods

### Procedures

Ethics approval for this study was obtained from the School of Pharmacy ethics committee at the Lebanese International University. All data were collected conveniently via a Google Form link during April 2023. The research approached some students, who were asked to forward the link to other university students they know. Inclusion criteria for participation included being of a resident and citizen of Lebanon of adult age. After providing digital informed consent, participants were asked to complete the survey, which were presented in a pre-randomised order to control for order effects. The survey was anonymous and participants completed the survey voluntarily and without remuneration [23].

### Participants

A total of 684 Lebanese young adults enrolled in this study with a mean age of 21.74 years (*SD* = 4.30), 65.6% females, 98.1% with a university level of education and 51.8% living in rural areas.

### Measures

#### Meaning in Life Questionnaire (MLQ)

The MLQ consists of 10 items and two subscales, each encompassing 5 items [1]. The first subscale is the Presence of meaning subscale (MLQ-P) which measures a person’s perception of the extent to which their life is meaningful [17]. The items which comprise the MLQ-P include “I understand my life’s meaning.” and “My life has a clear sense of purpose.” [1] The Search for meaning subscale (MLQ-S) is the second subscale which measures a person’s drive to find meaning in life [17]. Some of the items within the MLQ-S include “I am looking for something that makes my life feel meaningful.” and “I am always looking to find my life’s purpose.” [1] All MLQ items are scored on a Likert scale that ranges from 1 (*absolutely untrue*) till 7 (*absolutely true*), with higher scores identifying greater levels on the meaning in life construct [16].

#### The depression, anxiety, and stress scale (DASS-8)

The DASS-8, consisting of 8 items, is an abridged version of the DASS-21. The eight items are divided into three subscales, specifically 3 items for depression (e.g., “felt down hearted and blue”), 3 for anxiety (e.g., “felt scared without reason”), and 2 for stress (e.g., “was using a lot of my mental energy”) [24, 25]. The DASS-8 includes a Likert scale that ranges from 0 (did not apply to me at all) to 3 (applied to me very much or most of the time) [24]. The cumulative score of the DASS-8 ranges from 0 to 24, whereas the subscale scores range from 0 to 9 for each of the depression and anxiety subscales, and 0 to 6 for the stress subscale [25], with higher scores reflecting greater levels of symptoms (ω = 0.90 / α = 0.90).

#### Oviedo grit scale (EGO)

Validated in Arabic, the EGO consists of 10 items that measure grit, or one’s passion and perseverance that allows them to achieve goals in the face of adversity (i.e. “Although the results seem far off, I persist in the task”) [26]. The items are scored on a 5-point Likert scale that ranges from 1 (*totally disagree*) to 5 (*totally agree*) (ω = 0.95 / α = 0.93) [26].

#### Demographics

Participants were asked to provide their demographic details consisting of age and sex.

### Analytic strategy

#### Confirmatory factor analysis

Confirmatory factor analysis was conducted on the whole sample (*N* = 684) to test the original bi-dimensional structure of the MLQ. The CFA was performed using RStudio (Version 1.4.1103 for Macintosh) (R [27]), and the Lavaan [28] and semTools [29] packages. We used the weighted least squares means and variance adjusted (WLSMV) estimation method, which is more appropriate for ordinal data. A previous study suggested that the minimum sample size to conduct a confirmatory factor analysis ranges from 3 to 20 times the number of the scale’s variables [30]. Therefore, we assumed a minimum sample of 200 participants needed to have enough statistical power based on a ratio of 20 participants per one item of the scale, which was exceeded in this subsample. Our intention was to test the original bi-dimensional MLQ model. Parameter estimates were obtained using the maximum likelihood method and fit indices. Additionally, evidence of convergent validity was assessed using the Fornell-Larcker criterion, with average variance extracted (AVE) values of ≥ 0.50 considered adequate [31, 32].

#### Gender invariance

To examine gender invariance of MLQ scores, we conducted multi-group CFA using the total sample [33]. Measurement invariance was assessed at the configural, metric, and scalar levels [34]. We accepted ΔCFI ≤ 0.010 and ΔRMSEA ≤ 0.015 or ΔSRMR ≤ 0.010 as evidence of invariance [35].

#### Reliability and concurrent validity

Composite reliability in both subsamples was assessed using McDonald’s ω and Cronbach’s α, with values greater than 0.70 reflecting adequate composite reliability [36]. The MLQ score was normally distributed since its skewness ( = − 0.589) and kurtosis ( = − 0.145) values varied between ± 1. To assess convergent and criterion-related validity, we examined bivariate correlations between MLQ scores and those on the additional measures included in the survey (psychological distress and grit) using the total sample. Correlation coefficients values ≤ 0.10 were considered weak, ∼ 0.30 were considered moderate, and ∼ 0.50 were considered strong correlations [37].

## Results

### Confirmatory factor analysis

CFA indicated that fit of the bi-dimensional model of 10-item MLQ scores was inadequate: χ^2^/df = 50.91/13 = 3.92, RMSEA = 0.152 (90% CI 0.141, 0.163), SRMR = 0.099, CFI = 0.699, TLI = 0.602.

To improve this original model, which yielded relatively inadequate fit, we examined the modification index (MI). More specifically, the MI provide an estimate increase in the chi-square for each parameter if it were to be freed [38]. The MI outlined that the item 10 cross-loaded to both MLQ-P and MLQ-S factors. Accordingly, a modified model considering this cross-loading was created, by omitting item 10. Firstly, compared to the original model, the modified version demonstrated a lower robust chi-square (i.e., χ2 = 571.935 and χ2 = 262.087, respectively, with all *p* < .0001). As noted in previous studies [39] a low chi-square value relative to the degrees of freedom indicates a good model fit. Although this second model displayed significant increase in fit, compared to the original model, it still failed to fall within the acceptable range with a CFI of 0.853, a TLI of 0.796, a SRMR of 0.066, and RMSEA of 0.115 [90% CI of RMSEA (0.103, 0.128)].

Consequently, after a second examination of the MI pointed out item 9 also cross-loaded on both MLQ-P and MLQ-S factors, a third model was created by omitting items 9 and 10. Compared to the previous models, this final model displayed a gradual increase among all fit indices with a lower robust chi-square (i.e., χ2 = 69.367 with *p* < .0001), a greater CFI of 0.965, a TLI of 0.949, RMSEA of 0.062 [90% CI of RMSEA 0.047, 0.078] and a SRMR of 0.036. Reliability was good for the Search (ω = 0.89 / α = 0.89) and Presence (ω = 0.88 / α = 0.87) subscales.

Standardized factor loadings for the final bi-dimensional 10- and 8-item models of the MLQ can be found in Table 1.

**Table 1:** Standardized *of Factor Loadings from the Confirmatory Factor Analysis*

Item	Factor 1	Factor 2
**Model 1: 10-item MLQ**		
1. I understand my life’s meaning		0.78
2. I am looking for something that makes my life feel meaningful.	0.76	
3. I am always looking to find my life’s purpose.	0.82	
4. My life has a clear sense of purpose.		0.82
5. I have a good sense of what makes my life meaningful.		0.94
6. I have discovered a satisfying life purpose.		0.69
7. I am always searching for something that makes my life feel significant.	0.88	
8. I am seeking a purpose or mission for my life.	0.88	
9. My life has no clear purpose		− 0.23
10. I am searching for meaning in my life	0.45	
**Model 2: 8-item MLQ**		
1. I understand my life’s meaning		0.78
2. I am looking for something that makes my life feel meaningful.	0.72	
3. I am always looking to find my life’s purpose.	0.79	
4. My life has a clear sense of purpose.		0.80
5. I have a good sense of what makes my life meaningful.		0.92
6. I have discovered a satisfying life purpose.		0.69
7. I am always searching for something that makes my life feel significant.	0.88	
8. I am seeking a purpose or mission for my life.	0.85	

### Gender invariance

All indices suggested that configural, metric, and scalar invariance was supported across gender (Table 2). The Student t test results showed that no significant difference was found in terms of MLQ scores between females (*M* = 39.79, *SD* = 10.21) and males (*M* = 40.57, *SD* = 10.14) in the total sample, *t*(682) = 0.958, *p* = .338.

**Table 2:** Measurement Invariance across Gender in the total sample

Model	χ²	df	CFI	RMSEA	SRMR	Model Comparison	Δχ²	ΔCFI	ΔRMSEA	ΔSRMR	Δdf	*p*
Model 1: 10-item MLQ												
Configural	405.12	90	0.744	0.125	0.102							
Metric	395.12	76	0.751	0.130	0.101	Configural vs. metric	17.169	0.007	0.005	0.001	8	0.028
Scalar	405.12	84	0.744	0.125	0.102	Metric vs. scalar	17.169	0.007	0.005	0.001	8	0.028
Model 2: 8-item MLQ												
Configural	91.321	50	0.971	0.049	0.040							
Metric	82.135	44	0.973	0.050	0.039	Configural vs. metric	9.186	0.002	0.001	0.001	6	0.163
Scalar	91.321	50	0.971	0.049	0.040	Metric vs. scalar	9.186	0.002	0.001	0.001	6	0.163

Note. CFI = Comparative fit index; RMSEA = Steiger-Lind root mean square error of approximation; SRMR = Standardised root mean square residual

### Concurrent validity

Higher meaning in life total scores were associated with lower psychological distress (*r* = − .18; *p* < .001) and higher GRIT (*r* = .56; *p* < .001). The Search subscale was significantly and positively associated with higher GRIT (*r* = .48; *p* < .001) but not psychological distress (*r* = .01; *p* = .895), whereas the Presence subscale was significantly associated with higher GRIT (*r* = .57; *p* < .001) and lower psychological distress (*r* = − .31; *p* < .001).

## Discussion

The main goal of this study was to evaluate the psychometric characteristics of the Arabic version of the Meaning in Life Questionnaire (MLQ) when used with a sample of non-clinical Lebanese young adults who speak Arabic. Statistically, the scale demonstrated high reliability and offered support for its factorial and concurrent validities. It also showed consistent findings between males and females, showing scalar invariance among genders. As a result, these findings strongly indicate that the MLQ can be a dependable and valid instrument for assessing the presence of meaning as well as search for meaning in a sample of Arabic-speaking Lebanese young adults.

Based on the findings from the CFA, it is evident that the original, bi-dimensional model of the scale exhibited inadequate fit indices. The shortcomings of the original MIL model in the present sample could stem from several factors, including response bias. Response bias occurs when certain participants report false or inaccurate information on an assessment tool, thereby influencing the validity of the results [40]. This bias is prevalent in studies that utilize translated instruments and cross-cultural research, where cultural influences can impact participants’ answers [40]. A second reason possibly contributing to the inadequacy of the original MIL model in this study could be differential item functioning. This phenomenon arises when an item on a scale behaves differently in the original version compared to the translated version [41]. Another potential factor to consider is sampling error, which is common in any factor analysis [42]. In other words, items that exhibit weak factor loading within one set of data may not demonstrate the same pattern of weak factor loadings when the analysis is conducted with a different sample [42].

Considering these possible limitations, a modified model excluding item 10 (‘I am searching for meaning in my life.’) was then evaluated, considering that item 10 cross-loaded onto both MLQ-P and MLQ-S, yet this model still fell short of meeting the acceptable range of fit indices. Item 9 (‘My life has no clear purpose.’), which also cross-loaded into both MLQ subscales, was also excluded, yielding a final model with improvement across all fit indices. Thus, the 2 items were removed, leading to a better model fit with an 8-item MLQ compared to the 10-item MLQ, still revealing two factors with adequate reliability for the total score and the two. Upon further scrutiny of the factor loadings, it can be seen that, in both samples, fair to good values of resulted with the remaining 8 items of the MLQ, with all items presenting factor loadings higher than 0.7, except for item 6 which had a value of 0.69. Items that loaded into the Search subscale included items 2, 3, 7 and 8, while those that loaded into the Presence subscale included items 1, 4, 5, and 6. One tentative explanation for why items 9 and 10 were excluded can be the cultural biases that come with cross-cultural research. Although an instrument has been translated and back translated by experts, cultural interpretation of the items of the scale as well as one’s familiarity with the scale can lead to different patterns of response among participants from different cultures [43]. It is noteworthy to mention that while items on scale may be considered linguistically and functionally equivalent across different languages or cultures, they may still carry varying degrees of relevance to individuals from different backgrounds [43]. This variation can be attributed to the unique cultural and historical contexts that shape how participants understand and interpret concepts [43]. Essentially, certain themes may resonate more strongly with individuals within specific cultural or linguistic groups, leading to differences in the perceived significance of items on a scale [43].

Although other translated versions of the MLQ did not require the deletion of items, two factors still resulted, which is concordant with those found in other studies [1, 15–17]. Therefore, a bi-dimensional model is common for the MLQ, measuring two aspects of meaning in life, presence of and search for meaning., Given the limited psychometric data available on the MLQ in the Arabic-speaking world, our study raises the need to re-evaluate the factor structure of the Arabic MLQ using all items in larger and more representative samples of individuals from different Arab countries.

In line with other studies [1, 16], the two-factor model of the MLQ demonstrated gender invariance on three levels (Configural, Metric and Scalar). In other words, no significant gender-based distinctions were observed in the MLQ scores, affirming that the two-factor structure remained analogous for men and women. The DASS-8 and GRIT were used for the evaluation of the concurrent validity of the Arabic version of the MLQ. The results revealed a significant inverse relationship between higher meaning in life total scores and levels of psychological distress, alongside a positive association with grit. Prior research corroborates these results, indicating that heightened scores in meaning in life are consistently linked with reduced levels of psychological distress, anxiety, and depression [44]. Moreover, individuals reporting a robust sense of meaning in life tend to exhibit greater mental well-being compared to those with a weaker sense of meaning [45]. Regarding grit, empirical evidence demonstrates that individuals with greater sense of meaning in their lives tend to have elevated levels of grit [46]. Furthermore, and more specifically, the Presence subscale of the MLQ correlated with lower psychological distress. This is concordant with the findings of other studies that showed a positive relationship between the MLQ-P and general wellbeing [15] and mental health [16], and an inverse relationship with depression and negative affect [15]. This can be attributed with the idea that having meaning in one’s life is a sign of healthy psychological functioning and is attributed to wellbeing [16]. The positive correlation between the total MLQ score and the DASS-8 in the present study is further proof of this relationship. On the contrary, no significant relationship was found between the MLQ-S and psychological distress, being in line with some of the existing literature that state that searching for meaning in life does not relate to wellbeing nor to depression [47]. Other studies, however, point out that a negative association exists between searching for meaning and general mental health [16]. It is assumed that searching for meaning can be seen as problematic, being in line with searching for a source of wellbeing [16]. Additionally, both the MLQ-P and MLQ-S were seen to positively correlate with higher levels of grit. This can be attributed to the notion that people with adequate meaning in their life might be more resourceful in the face of adversity [48]. Similarly, it was found that grit was associated with higher presence of and search for meaning in life, theorizing that those with high levels of grit tend to realize the meaning in their lives and be driven to continuously find meaning; thus, putting effort in their commitment to life-long plans and goals [47]. However, some studies have found an inverse relationship between search for meaning and grit because young adults might be in a phase in their lives trying to experiment and explore different life commitments and roles to better understand their own identity and life goals [48]. Therefore, additional research is required to better understand the association between the meaning in life construct, psychological distress as well as grit.

More importantly, this study lacks an examination of measurement invariance between the Arabic version of the MLQ and the original versions of the scale. This step would be essential in determining whether the MLQ sustains measurement equivalence across cultures. As far as our scholarly inquiry extends, there is a dearth of cultural adaptation studies concerning measures of meaning in life that have investigated whether the translated MLQ accurately captures the same construct as the original measure. The absence of such an investigation into the measurement equivalence of the MLQ across diverse cultural contexts further underscores the necessity for such an investigation. Conducting such analyses would yield valuable insight into the cross-cultural validity and reliability of measures assessing meaning in life [49].

### Study limitations

The current study includes some limitations that should be considered and improved upon in future research. Firstly, the gathered sample might not be representative of the entire Lebanese population considering the recruitment method used. Furthermore, it is important to note that while this study may have demonstrated a good model fit based on the data that was gathered, this is not indicative that this model is the optimal one for the Arabic version of the MLQ. Therefore, future research might be able to identify another good model fit for the scale. Moreover, since the MLQ is a self-report measure, further studies should look into behavioral observations and longitudinal studies that could further clarify the concept of the meaning in life construct [1].

## Conclusion

The outcomes of the current study grants evidence for the psychometric soundness of the Arabic version of the MLQ; hence, affirming its appropriateness for the assessment of the presence of meaning and search for meaning in Lebanese young adults. With the provision of the Arabic version of the MLQ, we anticipate a more extensive examination of relationships and correlations between the meaning in life construct and diverse psychopathological phenomena and sociodemographic factors within a cultural and linguistic framework. Furthermore, this tool is anticipated to facilitate and promote international comparative studies and research collaborations, particularly among Arab countries.

## Data Availability

All data generated or analyzed during this study are not publicly available due to restrictions from the ethics committee, but are available upon a reasonable request from the corresponding author (SH).
